# Durability and extent of protection of SARS-CoV-2 antibodies among patients with COVID-19 in Metro Manila, Philippines

**DOI:** 10.3389/fimmu.2023.1190093

**Published:** 2023-06-28

**Authors:** Ma. Liza Antoinette M. Gonzales, Leonila F. Dans, Carol Stephanie C. Tan-Lim, Elenore Uy, Eva Cutiongco-dela Paz, Maria Vanessa V. Sulit, Marissa M. Alejandria, Mary Ann D. Lansang, Antonio L. Dans, Melissa A. Dator, Cynthia P. Cordero, Gina F. Pardilla

**Affiliations:** ^1^ Department of Pediatrics, College of Medicine, University of the Philippines Manila, Manila, Philippines; ^2^ Department of Clinical Epidemiology, College of Medicine, University of the Philippines Manila, Manila, Philippines; ^3^ Asia-Pacific Centre for Evidence-Based Healthcare, Manila, Philippines; ^4^ Institute of Human Genetics, National Institutes of Health, University of the Philippines Manila, Manila, Philippines; ^5^ Institute of Clinical Epidemiology, National Institutes of Health, University of the Philippines Manila, Manila, Philippines; ^6^ College of Medicine, University of the Philippines Manila, Manila, Philippines; ^7^ Manila Health Department Delpan Evacuation Center Quarantine Facility, Manila, Philippines

**Keywords:** antibody, humoral response, SARS-CoV-2, COVID-19, reinfection

## Abstract

**Introduction:**

Information on the magnitude and durability of humoral immunity against COVID-19 among specific populations can guide policies on vaccination, return from isolation and physical distancing measures. The study determined the durability of SARS-CoV-2 antibodies after an initial infection among Filipinos in Metro Manila, Philippines, and the extent of protection SARS-CoV-2 antibodies confer against reinfection.

**Methods:**

We conducted a cohort study to monitor the antibody levels of patients diagnosed with COVID-19. Receptor-binding domain (RBD)-specific antibodies were measured at Days 21, 90, 180, 270 and 360. Antibody levels were reported as geometric mean titers (GMT) with geometric standard deviation (GSD). Differences in GMT were tested using Friedman test and Kruskal Wallis test, with Bonferroni multiple comparisons procedure. Adjusted hazard ratios on the development of probable reinfection were estimated using Cox proportional models.

**Results:**

There were 307 study participants included in the study, with 13 dropouts. Study participants received SARS-CoV-2 vaccines at varying times, with 278 participants (90.5%) fully vaccinated by the end of study. The GMT of the study cohort increased over time, from 19.7 U/mL (GSD 11) at Day 21; to 284.5 U/mL (GSD 9.6) at Day 90; 1,061 U/mL (GSD 5.3) at Day 180; 2,003 U/mL (GSD 6.7) at Day 270; and 8,403 U/mL (GSD 3.1) at Day 360. The increase was statistically significant from Day 21 to Day 90 (p<0.0001), Day 90 to Day 180 (p=0.0005), and Day 270 to Day 360 (p<0.0001). Participants with more severe initial infection demonstrated significantly higher antibody levels compared to those with milder infection at Day 21. Sixty-four patients had probable COVID-19 reinfection (incidence of 20.8%, 95% CI 16.4, 25.8%). The GMT of these 64 patients was 411.8 U/mL (GSD 6.9) prior to the occurrence of the probable reinfection. Majority (87.5%) were fully vaccinated. Antibody titers significantly affected the risk of developing reinfection, with adjusted hazard ratio of 0.994, 95% CI 0.992-0.996, p<0.001.

**Conclusion:**

Antibody levels against SARS-CoV-2 increased over a one-year follow-up. Higher antibody levels were observed among those with more severe initial infection and those vaccinated. Higher antibody levels are associated with a lower risk of probable reinfection.

## 1 Introduction

Coronavirus disease 2019 (COVID-19) is a global pandemic that has caused tremendous health and socioeconomic consequences in the Philippines. As of March 2023, the Philippines has recorded over 4.08 million cases and over 66,118 deaths due to COVID-19. The Philippines’ poverty incidence rose to 23.7% in 2021, compared to 21.1% in 2018. Millions of Filipinos were unemployed, with the poor and marginalized sectors suffering most from the pandemic (1).

An important factor in controlling the spread of the infection and reinstating normal societal activities is determining what proportion of the population have developed antibodies (seroprevalence of the disease), and understanding whether the development of antibodies translates to immunity against subsequent infection in the long-term.

Several studies that monitored the long-term course of humoral immune response among those naturally infected with SARS-CoV-2 have shown a gradual decrease in receptor-binding and serum neutralizing antibody titers at varying time periods after infection (2–5).

A living review summarized the variation in antibody response to COVID-19 infection by age, sex, race, comorbidities and disease severity. Severe disease was associated with a more robust antibody response, with higher total antibody levels and neutralizing antibody capacity. Severe disease was also associated with a longer duration of detectable antibodies. Studies generally did not find a significant variation in antibody levels by age and sex. Evidence is unclear whether comorbidities are associated with antibody variation. In terms of variation of antibody levels by race or ethnicity, results suggest that non-Caucasians may exhibit higher antibody levels (3).

COVID-19 reinfection is well documented, and occurs when a person who has recovered from a previous SARS-CoV-2 infection becomes infected again. One study in the USA reported an incidence rate of 0.35 cases per 1,000 person-days among healthcare workers (6). Another study in India reported an incidence density of reinfection of 7.26 per 100 person-years (7).

The relationship between antibody levels and SARS-CoV-2 reinfection is a major area of clinical and public health interest. A case-control study done among unvaccinated individuals found that increasing anti-spike levels were associated with reduced risk of reinfection (odds ratio [OR] 0.63, 95% confidence interval [CI] 0.47 to 0.85). Using live virus microneutralization tests, titers >40 were associated with protection against reinfection. For pseudovirus microneutralization, titers>100 were associated with protection against reinfection (8).

In the Philippines, two studies evaluating COVID-19 seroprevalence have been published. One study determined seroprevalence (seropositivity defined as total SARS-CoV-2 immunoglobulin [Ig] ≥1 AU/mL) prior to the national vaccination program. The seroprevalence rates were 11.3% from May to July 2020, 46.8% from August to September 2020, 46% from December 2020 to January 2021, and 44.6% in March 2021 among residents in the city of Manila (9). Another study determined seroprevalence (seropositivity defined as receptor-binding domain [RBD]-Ig ≥0.8 U/mL) from June to December 2021, which coincided with the vaccine roll-out of the country in March 2021. The seroprevalence of the study population, which consisted of faculty, staff and students in a private tertiary university, ranged from 28.8% to 65.1%. The seropositive rate showed an increasing trend during the 7-month study period (10).

There are currently no studies among Filipinos that systematically monitors the quantitative antibody levels over a long-term period. Information on the timing, magnitude, and durability of humoral immunity among Filipinos is essential to guide the deployment of vaccine stocks, and can help guide strategies for returning from isolation and relaxing physical distancing measures. This study aimed to determine the durability of SARS-CoV-2 antibodies over a period of one year and the extent of protection these antibodies confer against reinfection among patients diagnosed with COVID-19. Specifically, we aimed to describe the pattern of antibody levels according to severity of initial COVID-19 infection, determine the incidence of reinfection among previously diagnosed COVID-19 patients, and determine if SARS-CoV-2 antibodies are protective against future infection

## 2 Methods

### 2.1 Study design

We conducted a cohort study to monitor the antibody levels of patients diagnosed with COVID-19. We followed up these patients to determine if there was reinfection within the first year after initial infection.

### 2.2 Study setting

We identified potential study participants from various COVID-19 hospitals and quarantine facilities in Metro Manila. We also invited potential participants by means of posters disseminated in social media platforms. We conducted the study remotely from the University of the Philippines, Manila from March 6, 2021 to July 12, 2022.

### 2.3 Study population

Patients who met the following eligibility criteria were enrolled to the study: 1) adult (≥18 years old); 2) diagnosed with COVID-19 through reverse transcription polymerase chain reaction (RT-PCR), including patients with asymptomatic, mild, moderate, severe or critical disease; 3) within 21 days since onset of symptoms (if symptomatic) or since RT-PCR positivity (if asymptomatic); 4) owned a mobile phone; 5) permanent address within Metro Manila; and 6) able to provide informed consent.

Due to anticipated changes in the circulating antibody levels, participants who received or intended to receive convalescent plasma or intravenous immunoglobulin during the follow-up and monitoring period were excluded.

Participants who received COVID-19 vaccine prior to enrollment were excluded from the study. However, due to ethical reasons, study participants who subsequently received the vaccine were still included in the study follow-up and determination of antibody levels.

### 2.4 Study procedures

Participants were followed up for one year, counting from the first day that they showed symptoms of COVID-19 or the day of RT-PCR positivity for asymptomatic patients.

#### 2.4.1 Remote coordination of study activities during the COVID-19 pandemic

The study researchers underwent training on Good Clinical Practice, study-specific consent process and documenting consent, and study-specific conduct of interviews of study participants prior to the start of study implementation. Study researchers operated from a virtual study hub, interacting with study participants through phone calls. The researchers performed eligibility screening, informed consent process, study data collection (at enrollment and follow-up), scheduling and coordination of study-related diagnostic tests, and tracking of patient location throughout the study. To minimize the risk of infection transmission, face-to-face interactions was limited to healthcare workers wearing the appropriate personal protective equipment and directly in charge of the clinical care of the study participants.

Third-party service providers were tapped to facilitate collection of specimens from the participants at the isolation center, at their respective residences or work place, or at barangay health centers, or at the nearest branch of the designated diagnostic laboratory, depending on the preference of the study participants. All collected blood specimens were transported to the accredited diagnostic laboratory who carried out the tests according to the manufacturer’s recommendations.

**Figure 1:**
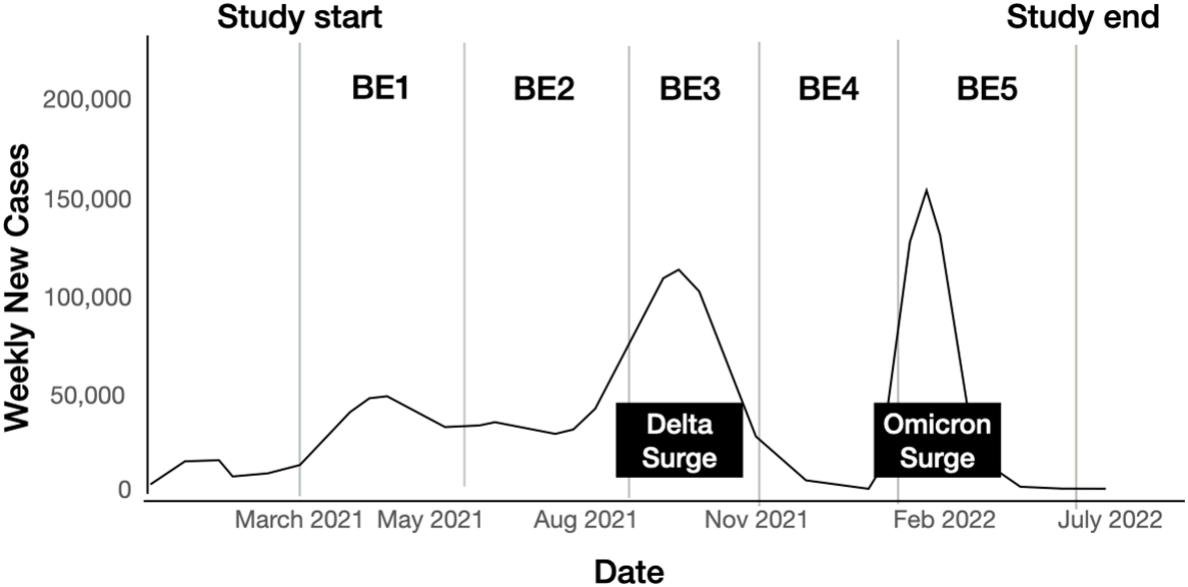
Epidemiological context in the Philippines and the timing of study implementation. (Image modified from the https://doh.gov.ph/covid19tracker)[12]. BE1 = first blood extraction at day 21, BE2 = second blood extraction at day 90, BE3 = third blood extraction at day 180, BE4 = fourth blood extraction at day 270, BE5 = fifth blood extraction at day 360.

Healthcare workers were involved in referring potentially eligible participants to the study staff. At the start of the study period, COVID-19 cases in Metro Manila were reaching 2,300 to 3,600 cases per day with variants P.1, P.3, B.1.1.7 and B.1.351 detected in the country (**Figure 1**) (11, 12). We anticipated that 20% of study participants would develop severe disease and require hospitalization. For this subset of patients, the study staff contacted the healthcare worker in the hospital to coordinate scheduled blood extractions and inquire about the results of diagnostic tests that were done as part of the study participant’s clinical care.

#### 2.4.2 Scheduled measurement of SARS-CoV-2 antibodies

We periodically measured the level of SARS-CoV-2 antibodies at Days 21, 90, 180, 270 and 360 (+/- 15 days) from onset of symptoms or date of RT-PCR positive test for asymptomatic patients, with allowable window period of +/-2 days for day 21 and +/- 15 days for the rest of the timepoints. This study used a laboratory-based semi-quantitative test, ECLIA (Elecsys^®^ Anti-SARS-CoV-2 S assay) to measure antibody levels. It detects the RBD-specific total antibody levels (IgG, IgA, IgM). The test is described in detail in Appendix 1. The lower limit of detection of the laboratory test used is 0.40 U/mL, while the upper limit of detection is 250 U/mL. For study participants who had results<0.40 U/mL, the result was recorded as 0.39 U/mL in the database in order to facilitate mathematical computation and data analysis. For study participants with results >250 U/mL, 10-fold dilution was performed to increase the upper limit of detection to 2,500 U/mL (13). Further dilution was performed as necessary to increase the upper limit of detection up to 250,000 U/mL.

#### 2.4.3 Monitoring for COVID-19 reinfection

During the one-year follow-up period, remote monitoring of study participants was done every two weeks to inquire the development of symptoms consistent with COVID-19 and result of the RT-PCR test or SARS-CoV-2 antigen test, if done.

An adjudication committee composed of five clinical epidemiologists, three of whom were also infectious disease specialist and one immunology-allergy specialist was formed. The committee classified participants who developed any COVID-19-like symptoms as confirmed, probable, possible or unlikely to have COVID-19 reinfection (defined in Appendix 2) based on the following: demographic information, relevant medical history, date of RT-PCR test indicating COVID-19 infection prior to enrollment, antibody levels before and after symptoms occurred, symptoms, duration of symptoms, history of exposure, type of occupation, RT-PCR test results and cycle threshold values (if available), and vaccination status. These information were collected by the study researchers through phone call to the study participants. The committee members were blinded to the identity of the patients and majority vote was followed.

### 2.5 Study variables

The following variables were collected at baseline COVID-19 disease severity, age, sex, co-morbidities. On follow-up SARS-CoV-2 antibody levels, incident COVID-19 (based on self-report of symptoms and laboratory results such as SARS-CoV-2 RT-PCR or antigen test, if available), vaccination status were recorded.

### 2.6 Biologic specimens

At each blood extraction, 10 ml of whole blood was drawn and placed in non-citrated vials for serum separation. One 5-ml vial each was collected for the following 1) laboratory-based antibody test, and 2) biobanking.

Blood specimens were stored for future testing, particularly PRNT once it is available. Serum samples were aliquoted in cryotubes and stored at the University of the Philippines National Institutes of Health (UP-NIH) at -70 to -80 degrees Celsius. We obtained written informed consent from the study participants for the storage of their blood samples for future testing. The blood samples collected in this study will be stored at the UP-NIH for a maximum of 25 years, according to the institution’s COVID-19 Samples Storage and Biobanking Policy.

### 2.7 Data collection and management

We used a secure data management software (Epidata) for study data collection. User access was restricted through user profiles designated according to user roles. Access to the system was given through individual accounts with password protection. A code assigned to each participant was used in the electronic questionnaires, which is only known to the researcher and the study staff. Electronic data was collated centrally and backed-up every day, at the end of the work day.

Data quality control was implemented by using both preventive and corrective actions. The electronic database, which captured the data electronically, was programmed with data quality rules that automatically perform calculations (e.g. age from birthdate), restrict allowable values to a specific range (e.g. a normal range of values for quantitative laboratory tests), use branching logic (e.g. *If yes* questions), and have mandatory items (i.e. empty response not allowed). At the end of the study, and before performing data analysis, frequency distribution of all variables was examined for out of range values and outliers. Data was also counterchecked from other data sources (e.g. medical records), as applicable. Furthermore, the electronic case forms of a random 10% of all the respondents underwent internal audit by an independent staff member who did not perform data collection to check the accuracy and completeness of the data.

### 2.8 Data analysis

Study data were processed using MS Excel and analyzed using STATA 17 software. Demographic, laboratory, and clinical data were presented using descriptive statistics. Mean with standard deviation (or median and IQR) was used to describe quantitative data. For qualitative data, frequencies were used. Antibody levels were reported as geometric mean titers (GMT) with geometric standard deviation (GSD) at each period of observation, as these are the recommended measures of location and dispersion for antibody titers (14). Antibody GMTs with GSD were also reported according to initial COVID-19 severity classification and the vaccination status of the participants.

Friedman test was used to compare GMTs across the 5 timepoints. If significant differences was found, pairwise sign test was done at 5% level of significance with adjustments using Bonferroni method ( α=0.05 divided by 10 pairwise comparisons). The adjusted alpha used was 0.005 and all p-values were compared with the adjusted alpha. Kruskal Wallis test was used to compare the GMTs based on severity classification. If significant difference was found, Dunn’s test was done at Bonferroni adjusted level of significance of 0.005 for severity classification ( α=0.05 divided by all 10 pairwise comparisons).

The incidence of reinfection was estimated at 95% confidence level. Unadjusted and adjusted hazard ratios for the effect of antibody levels on the development of probable reinfection were estimated using Cox proportional hazards model. Antibody level was treated as a continuous variable. The antibody level prior to the reinfection was used for those with probable reinfection. For those without probable reinfection, their GMT across the 5 timepoints were used. Hazard ratios were adjusted for possible confounders including age, sex, co-morbidities, and vaccination status.

### 2.9 Sample size computation

Liu et al. (15) reported a standard deviation of 246 IgG RU/ml for patients with COVID-19 infection on day 14. Using this standard deviation, 244 participants are needed to estimate the mean IgG titer at 99% level and 80% probability of achieving a target width of 88 RU/ml. The level of confidence was adjusted for multiple comparisons by the Bonferroni method since the mean titer will be estimated at 5 periods of observations (alpha=0.05/5 = 0.01).

Taking into consideration a possible dropout rate of 20%, this study targeted to recruit a total of 307 participants. Dropout is defined as a situation where all outcome data of the participant are missing after a certain timepoint. This includes mortality, withdrawal of consent, and loss to follow-up.

### 2.10 Ethical considerations

This study was conducted following the principles outlined in the Declaration of Helsinki, the WHO International Ethical Guidelines for Health-related Research Involving Humans, and the Philippines’ National Ethical Guidelines for Health and Health-Related Research. This research was reviewed and approved by the UP Manila Research Ethics Board. The study protocol was submitted to the UP Manila Institutional Biosafety and Biosecurity Committee for review and clearance. Ethics Review Board (ERB) approval was secured before the start of the study (UPMREB 2020-698-01).

## 3 Results

### 3.1 Study participants

From March 6 2021 to June 15 2021, a total of 536 participants were screened. Potential participants came from quarantine facilities (QF) and COVID-19 centers in Metro Manila. We also posted an infographic describing the objectives of the study and inclusion criteria in different social media platforms (i.e. Facebook, Instagram, Twitter and group chats) which included contact details of the research staff for anyone interested to participate. Of the 536 participants screened, 229 were excluded. The reasons for exclusion included no permanent address in Metro Manila (n=53), onset of symptoms beyond 21 days (n=21), receipt of SARS-CoV-2 vaccine or convalescent plasma (n=16), no mobile phone (n=7), no consent to participate (n=124), inability to have the first blood extraction done due to logistical difficulties (n=5) and mortality prior to the first blood extraction (n=3).

Of the 307 participants enrolled, 123 (40.1%) came from QFs and COVID-19 centers, while 184 (59.9%) were identified through social media. The participants were followed up for one year, with the end of follow-up period on July 12, 2022. Over the course of the study, there were four participants who died. Two participants died due to acute respiratory failure after the day 21 blood extraction. One participant developed respiratory failure from hospital-acquired pneumonia and the other one developed respiratory failure due to a mixed connective tissue disease that was diagnosed in 2006. At the time of demise, the SARS-CoV-2 RT-PCR test in the 2 participants taken 30 days and 39 days from the initial positive RT-PCR test, respectively were negative. Two participants died after the fourth (day 270) blood extraction timepoint. One participant had chronic kidney disease stage 5 secondary to chronic glomerulonephritis, requiring maintenance hemodialysis. She was reported to have missed dialysis sessions due to vascular access malfunction. At the time of demise, she had sudden onset of difficulty of breathing, for which she was brought to a hospital where sudden cardiac death was declared as her primary cause of death. Her last blood extraction was three months prior to her demise, with results of 6,527 U/mL. The other participant had no known co-morbid conditions but reportedly had cardiomegaly detected by chest radiograph 6.5 months after enrollment with no further work-up done. He developed a probable COVID-19 re-infection 7 months after enrollment, 4 months prior to his demise. The participant presented with mild symptoms and recovered after completion of home isolation. His last blood extraction was 2.5 months prior to his demise, with SARS-CoV-2 antibody levels of 6,545 U/mL. He was found dead at home with an unknown cause of death.

There were 9 patients who withdrew from the study. The reasons for withdrawal included refusal to have additional blood extractions done (4 participants), inability to contact the study participants (2 participants), maritime employment (2 participants) and difficulty in scheduling blood extractions due to work (1 participant).

**Figure 2:**
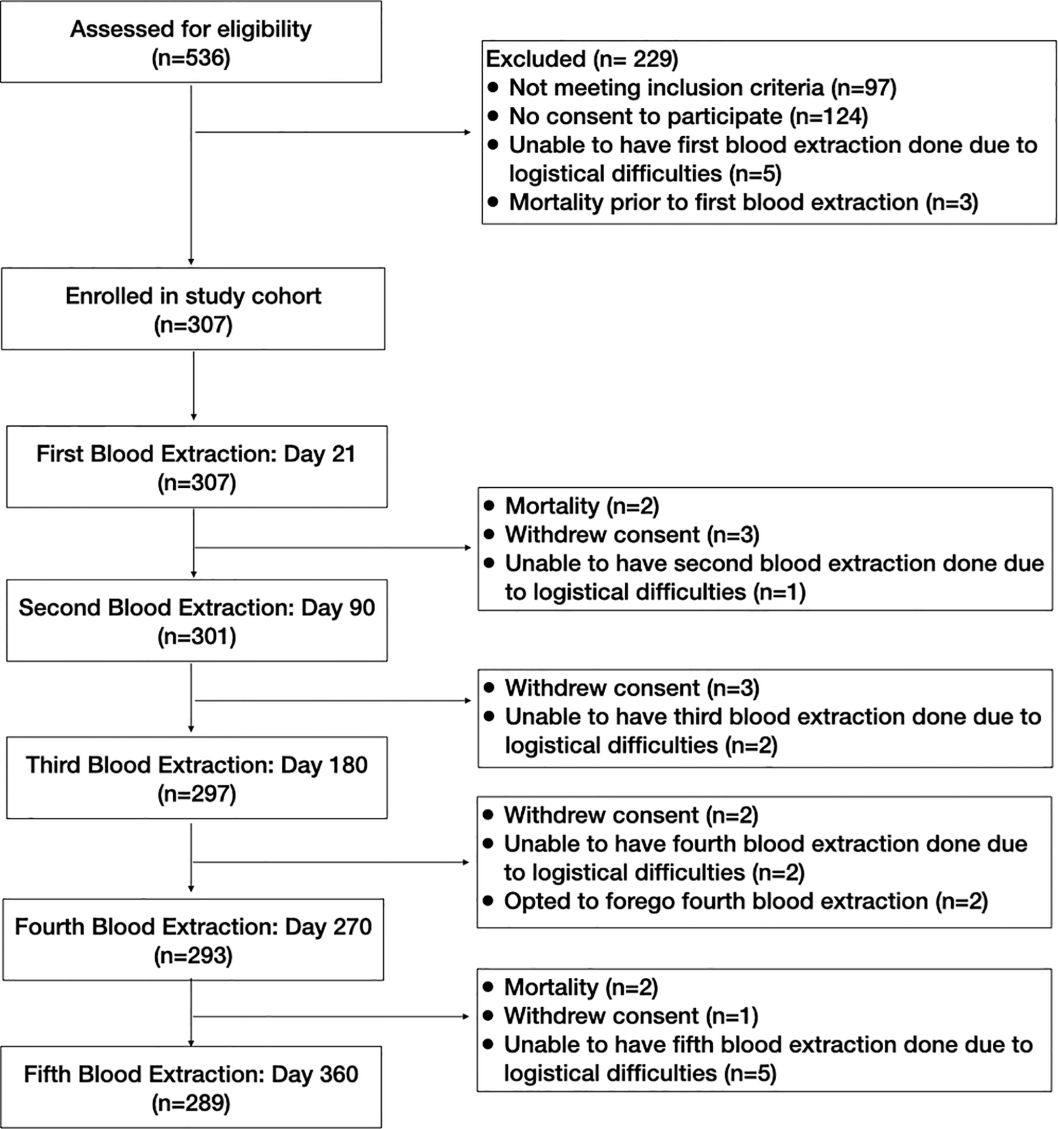
Flowchart of screening, enrollment and monitoring procedures.

The study participants’ flow diagram is shown in **Figure 2**. All 307 study participants underwent the first blood extraction. There were only 301 study participants who underwent the second blood extraction because 2 participants died, 3 participants withdrew consent, and 1 participant was unable to have the blood extraction done due to logistical difficulties. This one participant who had logistical difficulties for the second blood extraction was still considered to be enrolled in the study with a missing data point for the second blood extraction. Of the 302 study participants in the study cohort after the second blood extraction, 297 underwent the third blood extraction. There were 3 additional participants who withdrew consent, and 2 participants who were unable to have the third blood extraction done due to logistical difficulties. Of the 299 study participants in the study cohort after the third blood extraction, 293 underwent the fourth blood extraction. There were 2 additional participants who withdrew consent, and 2 participants who were unable to have the third blood extraction done due to logistical difficulties, and 2 participants who opted to defer the fourth blood extraction due to medical reasons. One had anemia, while the other participant was on blood thinners and had multiple hematomas. Of the 297 study participants in the study cohort after the fourth blood extraction, 289 underwent the fifth blood extraction. There were 2 participants who died, 1 participant who withdrew consent, and 5 participants who were unable to have the fifth blood extraction done due to logistical difficulties.

The baseline characteristics of the study participants are shown in **Table 1**. The median age was 36 years old (interquartile range [IQR] 19), with slightly more females (53.4%). Majority of the participants were classified to have mild COVID-19 infection (55.4%).

**Table 1:** Baseline demographic and clinical characteristics of the study participants, Metro Manila, Philippines (N = 307).

Variable	Frequency (%) N = 307
**Age**	Median 36 years (IQR 19)
19 to 59 years old	279 (90.9)
60 and above	28 (9.1)
Sex	
Male	143 (46.6)
Female	164 (53.4)
Severity	
Asymptomatic patients	78 (25.4)
Mild disease	170 (55.4)
Moderate disease	24 (7.8)
Severe disease	28 (9.1)
Critical Disease	7 (2.3)
**On immunosuppressants**	3 (1.0)
Co-morbidities	
Hypertension	63 (20.5)
Pulmonary diseases	38 (12.4)
Diabetes	30 (9.8)
Gastrointestinal disorders	17 (5.5)
Neurologic disorders	16 (5.2)
Dyslipidemia	7 (2.3)
Rheumatologic diseases	7 (2.3)
Oncologic disorders	5 (1.6)
Chronic kidney disease	4 (1.3)
Cardiac diseases	4 (1.3)
Endocrine diseases	4 (1.3)
Psychiatric diseases	3 (1.0)
Hematologic diseases	2 (0.7)

Of the 307 enrolled participants, 117 (38.1%) had co-morbidities. The most common co-morbidities were hypertension (20.5%), pulmonary diseases (12.4%) and diabetes mellitus (9.8%). Among the respiratory diseases, asthma was the most common (n = 27). Other respiratory diseases reported were interstitial lung disease, chronic obstructive pulmonary disease and tuberculosis. Other comorbidities were gastrointestinal disorders (e.g., cholelithiasis, ulcer, gastroesophageal reflux disease, fatty liver, hepatitis, Crohn’s disease) and neurologic conditions such as cerebrovascular accident, migraine, vertigo and Parkinson’s disease, pituitary macroadenoma and history of encephalitis. Chronic cardiac conditions identified were arrhythmia, mitral valve prolapse, and coronary artery disease. Some participants also had chronic kidney conditions (e.g., polycystic kidney, nephrolithiasis), five of whom were undergoing hemodialysis. Cases of neoplastic diseases in the cohort included breast, colorectal, prostate, nasopharyngeal cancer and chronic myelogenous leukemia.

There were no cases of HIV infection among the study participants. Three participants were on immunosuppressants for autoimmune conditions including primary macroadenoma, Sjogren’s disease and systemic lupus erythematosus.

### 3.2 Antibody levels of study participants

**Figure 3:**
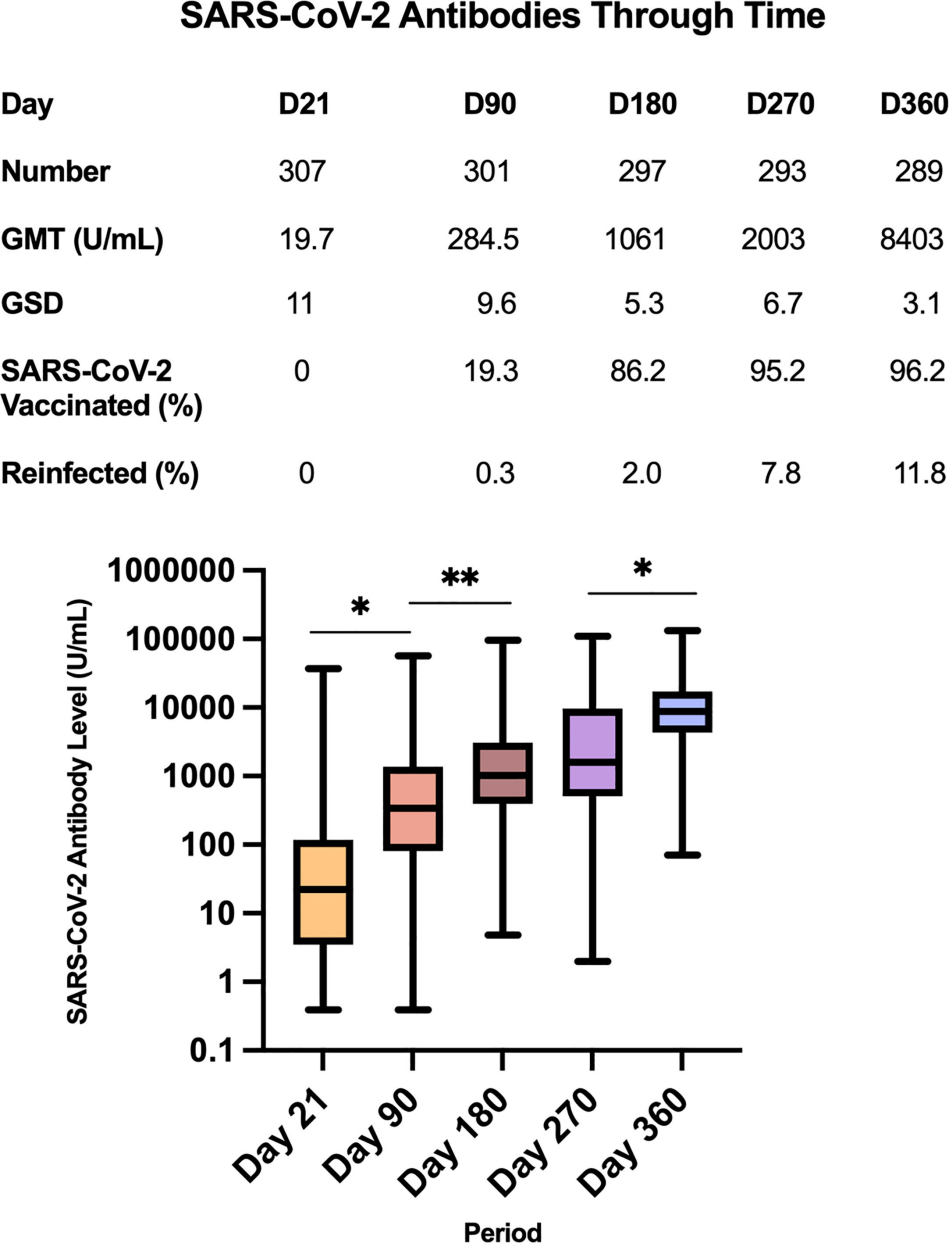
Geometric mean titers of SARS-CoV-2 antibodies (U/mL) of all study participants across the 5 timepoints *p=<0.0001, **p=0.0005.

The total RBD-specific immunoglobulin levels of the entire study cohort for each of the five blood extraction timepoints are shown in **Figure 3**. The GMT of the study cohort increased over time. At day 21, the GMT was 19.7 U/mL, with GSD 11 (n=307). At day 90, the GMT significantly increased to 284.5 U/mL (GSD 9.6; n=301), p=<0.0001. At day 180, the GMT was 1,061 U/mL (GSD 5.3, n=297). The increase from day 90 to day 180 was statistically significant (p=0.0005). At day 270, the GMT was 2,003 U/mL (GSD 6.7; n=293), although this increase from day 180 was not statistically significant (p=0.098). At day 360, the GMT significantly increased to 8,403 U/mL (GSD 3.1; n=289) compared to the day 270 GMT (p=<0.0001).

However, it should be noted that at Day 21, 22 study participants had antibody titers below the lower limit of detection (<0.40 U/mL). To facilitate data analysis, these were encoded as 0.39 U/mL. Hence the GMT of 19.7 U/mL at Day 21 is likely an overestimate of the actual value. At Day 90, only 7 study participants had antibody titers<0.40 U/mL. Similarly, the GMT of 284.5 U/mL at Day 90 is likely an overestimate of the actual value. There were no study participants with titers<0.40 U/mL on Days 90 to 360.

#### 3.2.1 SARS COV-2 antibody levels according to initial COVID-19 severity classification

**Figure 4:**
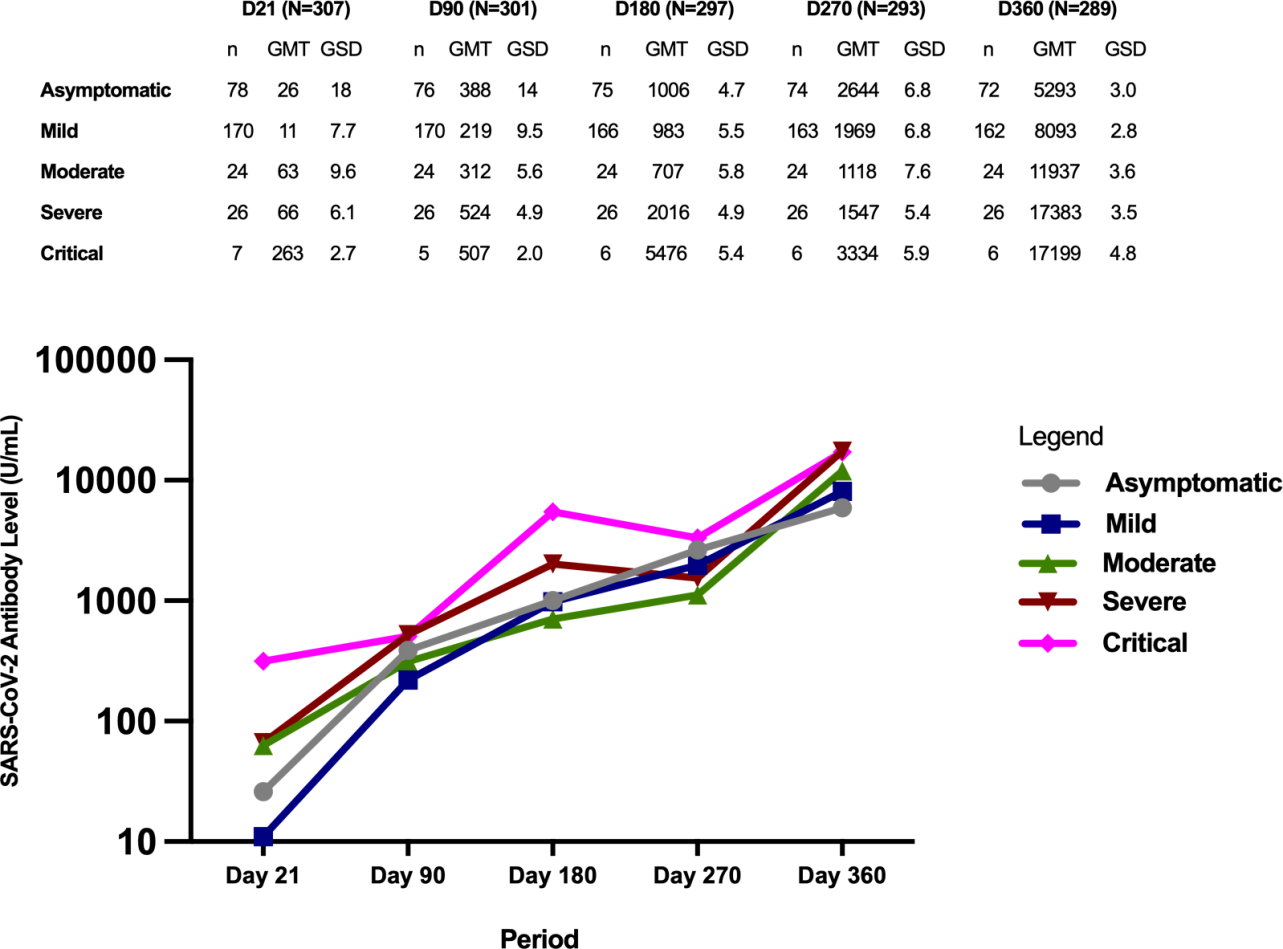
Geometric mean titers of SARS-CoV-2 antibodies (U/mL) according to initial COVID-19 disease severity classification.

**Figure 4** shows the antibody GMT according to initial COVID-19 disease severity classification. It can be observed that regardless of baseline severity classification, the antibody GMTs generally showed an increasing trend from Day 21 until Day 360.

At day 21, study participants with critical disease severity had significantly higher GMTs compared to those with mild disease (p=<0.0001) and asymptomatic disease (p=0.002). Those with severe disease and moderate disease also had higher antibody GMTs compared to those with mild disease (p=<0.001 for severe, p=0.0001 for moderate), and asymptomatic disease (but not statistically significant at p=0.008 for severe vs asymptomatic, p=0.019 for moderate vs asymptomatic). Similarly, although those with critical disease had higher antibody GMTs compared to those with moderate and severe disease, the difference did not reach statistical significance (p=0.072 for critical vs moderate, p=0.088 for critical vs severe).

The antibody GMTs of participants with severe and critical infection remained higher compared to those with asymptomatic, mild, and moderate infection on Day 90 and D180, but the differences were not statistically significant. Similarly, those with critical infection had higher antibody GMTs compared to the rest of the severity groups on Day 270, but the difference was not statistically significant.

The highest antibody GMTs were observed on Day 360 across all the severity groups. Those with more severe infection had higher GMTs compared to those with milder severity classification.

Among the 22 participants with titers<0.40 U/mL at Day 21, 11 had asymptomatic infection, 9 had mild infection, 1 had moderate infection, and 1 had severe infection. Of the 7 participants with titers<0.40 U/mL at Day 90, 3 had asymptomatic infection and 4 had mild infection.

#### 3.2.2 Subgroup analysis of antibody levels by vaccination status at the end of the study

The study participants received the SARS-CoV-2 vaccine at varying times. At the time of the day 90 blood extraction, 117 participants (38.1% of the entire study cohort) were partially vaccinated and 60 (19.5%) were fully vaccinated and by the end of the follow-up period (Day 360), 278 (90.5%) had been fully vaccinated, with 66 (21.5%) completing only the primary series, 209 (68%) receiving 1 booster dose, and 3 (1%) receiving 2 booster doses.

**Figure 5:**
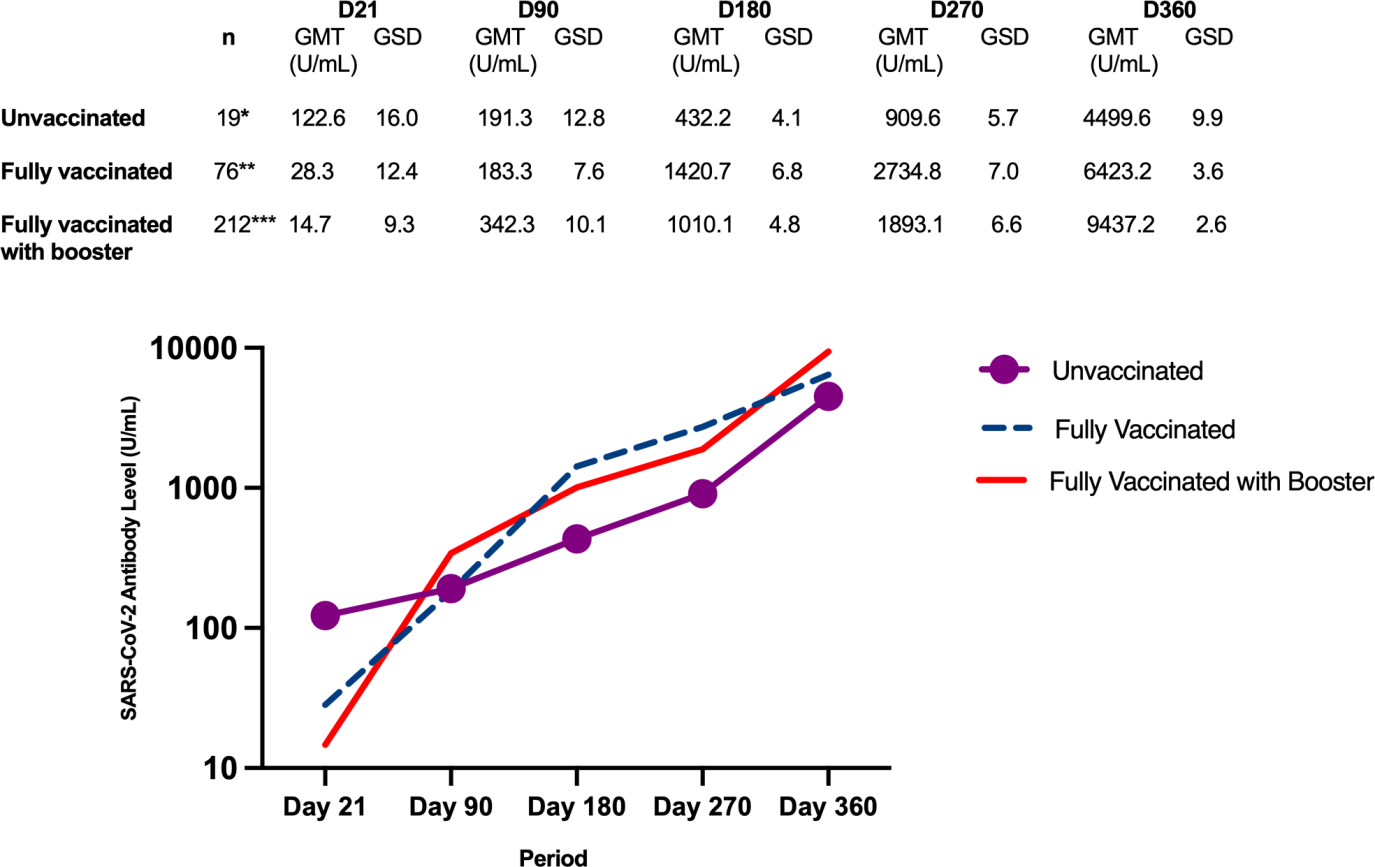
Geometric mean titers of SARS-CoV-2 antibody levels (U/mL) according to vaccination status at the end of the study. *Day 90 - 5 dropouts (n=14); Day 180 - 1 dropout and 1 missed blood extraction (n=12); Day 270 - 1 missed blood extraction (n=12); Day 360 - 1 dropout and 1 missed blood extraction (n=11). **Day 90 - 13 partially vaccinated and 13 fully vaccinated; Day 180 - 13 partially vaccinated and 54 fully vaccinated, 2 dropouts and 1 missed blood extraction (n=73); Day 270 – 68 fully vaccinated, 2 dropouts and 3 missed blood extractions (n=69); Day 360 – 66 fully vaccinated, 2 dropouts and 4 missed blood extractions (n=66). ***Day 90 – 104 partially vaccinated and 47 fully vaccinated, 1 missed blood extraction (n=211); Day 180 – 9 partially vaccinated, 202 fully vaccinated (n=212); Day 270 – 1 partially vaccinated, 155 fully vaccinated, 56 with booster; Day 360 – 212 with booster.

**Figure 5** shows the antibody GMT according to vaccination status at the end of the study. Regardless of baseline severity classification, the antibody GMTs generally showed an increasing trend from Day 21 until Day 360.

From Day 180 to 360, as more study participants received COVID-19 vaccine, the antibody GMTs of the fully vaccinated group and the booster group increased at a greater magnitude compared to those in the unvaccinated group. By the end of the study, when the majority of the participants had already received booster doses, the antibody GMT of the booster group was higher than the group that received only the primary series.

### 3.3 Reinfection in the study cohort

There were a total of 303 reports of COVID-19-like symptoms during the one year follow-up. Some participants had more than 1 report of COVID-19-like symptoms within the follow-up period. Of the 303 reports, 64 (21.1%) were adjudicated to have had probable COVID-19 reinfection, 101 with possible reinfection (33.3%), and 138 were unlikely to have had reinfection (45.5%). There were no confirmed COVID-19 reinfection because genomic testing could not be performed in the respiratory specimens taken from each infection episode. The incidence of probable COVID-19 reinfection in this cohort was 20.8% (95% CI 16.4 to 25.8%).

#### 3.3.1 Probable reinfection

There were 64 cases of probable reinfection occurring in 64 study participants. Six (9.4%) occurred during the Delta variant surge from August to October 2021 while 39 (60.9%) occurred during the Omicron variant surge from January to February 2022. The remaining 19 (29.7%) did not coincide with the observed surges in the Philippines.

The characteristics of the study participants with probable reinfection are shown in **Table 2A**. More than half (36 participants, 56.3%) of the probable cases of reinfection occurred among participants who previously had a mild COVID-19 infection. None of the participants who had previous critical infection had a probable reinfection. The most common co-morbidities of those who experienced probable reinfection were hypertension (23.4%) and diabetes mellitus (9.4%).

**Table 2:** Clinical and laboratory characteristics of the study participants with probable, possible and unlikely COVID-19 reinfection, Metro Manila, Philippines (N=262).

Variable	A. Probable reinfection n=64 Frequency (%)	B. Possible reinfection n=88 Frequency (%)	C. Unlikely reinfection n=110 Frequency (%)
Age, *years* ^a^	34 (IQR 16)	36 (IQR 14.5)	38 (IQR 16.8)
Sex			
Male	30 (46.9)	42 (47.7)	44 (40%)
Female	34 (53.1)	46 (52.3)	66 (60%)
Initial Severity Classification			
Asymptomatic patients	14 (21.8)	21 (23.9)	25 (22.7)
Mild disease	36 (56.3)	50 (56.8)	57 (51.8)
Moderate disease	9 (14.1)	8 (9.1)	10 (9.1)
Severe disease	5 (7.8)	8 (9.1)	15 (13.6)
Critical Disease	0	1 (1.1)	3 (2.7)
Co-morbidities			
Hypertension	15 (23.4)	13 (14.8)	24 (21.8)
Diabetes mellitus	6 (9.4)	4 (4.5)	6 (5.5)
Asthma	4 (6.3)	9 (10.2)	13 (11.8)
Cardiac conditions	3 (4.7)	1 (1.1)	1 (0.9)
Oncologic conditions	2 (3.2)	2 (2.3)	1 (0.9)
Gastrointestinal esophageal reflux disease	1 (1.6)	3 (3.4)	1 (0.9)
Allergic rhinitis	1 (1.6)	0 (0)	1 (0.9)
Chronic kidney disease	1 (1.6)	3 (3.4)	3 (2.7)
SARS-CoV-2 antibody level (U/mL)^b^			
Day 21	16.8 (9.8)	15.6 (12.6)	21.6 (11.4)
Prior to reinfection	411.8 (6.9)	501.0 (8.9)	197.8 (18.9)
Vaccination status^c^			
Unvaccinated	7 (10.93)	14 (13.9)	49 (35.5)
Partially vaccinated	1 (1.6)	6 (5.9)	14 (10.2)
Fully vaccinated (without booster)^d^			
Sinovac	32 (50)	40 (39.6)	40 (29.0)
Astra-Zeneca	7 (10.9)	4 (4.0)	4 (2.9)
Sputnik	4 (6.3)	0	7 (5.1)
Pfizer	4 (6.3)	4 (4.0)	15 (10.9)
Moderna	4 (6.3)	12 (11.9)	2 (1.4)
J&J	0	1 (0.9)	2 (1.4)
Fully vaccinated with Booster	5 (9.3)	20 (19.8)	5 (3.6)
History of exposure to COVID-19^e^			
Symptomatic close contact	33 (51.5)	20 (19.8)	6 (4.3)
High-risk employment^f^	6 (9.4)	5 (5.0)	4 (2.9)
None	25 (39.1)	76 (75.2)	128 (92.8)

a Median, interquartile range (IQR).

b Geometric mean titer (GMT), geometric standard deviation (GSD); N=101 cases for possible reinfection, N=138 cases for unlikely reinfection.

c At the time of report of COVID-19 symptoms, N=101 cases for possible reinfection, N=138 cases for unlikely reinfection.

d Sinovac - inactivated vaccine; Astra-Zeneca, Sputnik, Johnson & Johnson - adenovirus vector vaccine; Pfizer, Moderna - mRNA vaccines.

e N=101 cases for possible reinfection, N=138 for unlikely reinfection.

f High risk employment refer to frontline workers including healthcare workers and protective service workers (e.g. police).

The antibody GMT of participants with probable reinfection at the first blood extraction timepoint (day 21) was 16.8 U/mL (GSD 9.8, range<0.40 to 1,269). Prior to reinfection, the antibody GMT of the 64 participants with probable reinfection was 422.2 U/mL (GSD 6.3, range 1.98 to 34,570), with an average time interval from the date of antibody determination to the onset of symptoms of 53.8 days (SD 49.8).

Of the 64 probable reinfection cases, 56 were fully vaccinated, one was partially vaccinated and 7 were unvaccinated at the time of re-infection. Of the 56 fully vaccinated who developed probable reinfection, there were 5 who developed probable reinfection after receiving 1 booster dose. The most common vaccine received was an inactivated SARS-Co-V-2 vaccine, Sinovac (36 participants or 56.3%, of which 32 participants completed the primary series of two doses, while 4 participants received one additional mRNA booster vaccine dose).

A little more than half of the participants with probable reinfection had a history of exposure to a symptomatic close contact (51.5%). Thirty (46.9%) were diagnosed through a positive RT-PCR test, 14 (21.9%) were diagnosed through a positive SARS-CoV-2 antigen test, and 20 (31.2%) were diagnosed based on a spike in their SARS-CoV-2 antibody levels not otherwise explained by vaccination.

The timing of reinfection and outcomes are shown in **Table 3**. There was only 1 study participant who had a probable reinfection less than 3 months from the initial COVID-19 infection. This participant reported complete resolution of symptoms after the initial mild infection, but there was no documentation of a negative RT-PCR test. The antibody titer at Day 21 was 0.61 U/mL. This study participant presented with symptoms of cough, fever, fatigue, sore throat, and nasal congestion and had a positive repeat RT-PCR test taken 82 days after the initial infection. This participant was unvaccinated at the time of the probable reinfection.

**Table 3:** Timing and outcomes of the patients with probable reinfection, Metro Manila, Philippines (n-64).

Variables	Probable reinfection n=64 Frequency (%)	Antibody GMT U/mL (GSD)^a^
Timing of reinfection (from initial		
COVID-19 infection)**<**3 months	1 (1.6)	0.61
3 to<6 months	6 (9.4)	4.1 (9.7)
6 to<9 months	23 (35.9)	15.9 (6.8)
9 to 12 months	34 (53.1)	24.5 (11.3)
Severity classification of reinfection		
Asymptomatic	0	–
Mild	47 (73.4)	470.8 (9.0)
Moderate	16 (25)	638.0 (2.5)
Severe	1 (1.6)	269.8
Critical	0	–
Outcomes		
Recovered after home isolation	63 (98.4)	422.2 (6.3)
Hospitalized	1 (1.6)	269.8
Mortality	0	–

a Geometric mean titer (GMT), geometric standard deviation (GSD); Antibody levels obtained at Day 21 for timing of reinfection; Antibody levels obtained prior to reinfection report for severity classification and outcomes.

There were 6 participants (9.4%) who developed reinfection 3 to 6 months from the initial infection, of which 2 were unvaccinated, 1 was partially vaccinated, and 3 were fully vaccinated. The antibody GMT of the unvaccinated participants prior to infection was 58 U/mL (GSD 4.2) while the vaccinated participants had a GMT of 103.7 U/mL (GSD 4.1). There were 23 participants (35.9%) who developed probable reinfection 6 to 9 months from the initial infection, all of whom were fully vaccinated. Their antibody GMT prior to infection was 603.6 U/mL (GSD 6.1). Majority of the participants (34 participants or 53.1%) developed probable reinfection 9 to 12 months after the initial infection. Of the 34 participants, 4 were unvaccinated, 25 were fully vaccinated, and 5 had 1 booster dose. The antibody GMT of the unvaccinated participants prior to infection was 416.6 U/mL (GSD 2.1) while the vaccinated participants had a GMT of 522 U/mL (GSD 5.8). The average time to reinfection from initial infection among the 64 participants was 253 days (SD 56).

Majority (73.4%) had mild disease on reinfection. There were 16 participants (25%) with moderate disease and 1 participant (1.6%) with severe disease. The participant who developed severe disease upon reinfection needed hospitalization and oxygen support through nasal cannula. This participant was a 31-year-old female with diabetes mellitus and heart failure. This participant was fully vaccinated with Sinovac 3.5 months prior to reinfection, with no booster dose received. She was treated with remdesivir, dexamethasone, and enoxaparin, with improvement of symptoms and was discharged well after 11 days. Her antibody titers at the first extraction timepoint (Day 21) was 38.3 U/mL, while her antibody titers 45 days prior to the probable reinfection was 269.8 U/mL. In contrast, those with mild reinfection had antibody GMT of 470.8 U/mL (GSD 9.0) extracted on the average 58 days prior to the development of reinfection. Those with moderate reinfection had antibody GMT of 638.0 U/mL (GSD 2.5), extracted on the average 40 days prior to the development of reinfection. Majority of the participants (98.4%) with probable reinfection recovered after home isolation.

#### 3.3.2 Possible reinfection

There were 101 reports of COVID-19 like symptoms adjudicated as possible reinfection occurring in 88 study participants. There were 11 participants with 2 possible reinfection, and 1 participant with 3 possible reinfections over the one-year study period. Of the 101 cases, 15 (14.9%) occurred during the Delta variant surge from August to October 2021 while 53 (52.5%) occurred during the Omicron variant surge from January to February 2022. The remaining 33 (32.7%) did not coincide with the observed surges in the Philippines.

The characteristics of the study participants with possible reinfection are shown in **Table 2B**. Similar to the patients with probable reinfection, most (56.8%) of the possible cases of reinfection occurred among participants who previously had mild infection. The most common co-morbidities of those who experienced possible reinfection were hypertension (14.8%) and asthma (10.2%).

The antibody GMT of those with possible reinfection at the first blood extraction timepoint (day 21) was 15.6 U/mL (GSD 12.6). Of the 101 reports, 14 were unvaccinated at the time of possible reinfection, 6 were partially vaccinated, 61 were fully vaccinated, and 20 had 1 booster dose. Prior to reinfection, the antibody GMT of the unvaccinated participants was 141.5 U/mL (GSD 10.1), while the GMT of the vaccinated participants was 594.3 U/mL (GSD 8.1). The average time interval from the date of antibody determination to the onset of symptoms of 42.5 days (SD 31.3).

The most common vaccine received was an inactivated COVID-19 vaccine (Sinovac) in 54 cases or 53.5%, of which 40 completed the primary series while 14 received one booster vaccine dose. Of the 20 participants who received a booster dose, 8 participants received Pfizer mRNA vaccine, 7 participants received Moderna mRNA vaccine, 4 participants received an adenoviral vector vaccine (Astra-Zeneca), and 1 participant received an inactivated vaccine (Sinovac).Only 20 cases of possible reinfection (19.8%) had a history of exposure to a symptomatic close contact.

#### 3.3.3 Unlikely reinfection

There were 138 reports of COVID-19-like symptoms adjudicated as unlikely reinfection occurring in 110 study participants. There were 23 participants with 2 COVID-19-like events, 1 participant with 3 events, and 1 participant with 4 events, subsequently adjudicated as unlikely reinfection. Of the 138 reports, 39 (28.3%) occurred during the Delta variant surge from August to October 2021 while 6 (4.3%) occurred during the Omicron variant surge from January to February 2022. The remaining 93 (67.4%) did not coincide with the observed surges in the Philippines.

The characteristics of the study participants with unlikely reinfection are shown in **Table 2C**. Similar to the probable reinfection, most (51.8%) of the cases occurred among participants with previous mild infection. The most common co-morbidities were hypertension (21.8%) and asthma (11.8%).

The antibody GMT of those adjudicated as unlikely reinfection at the first blood extraction timepoint (day 21) was 15.6 U/mL (GSD 12.6). Prior to reinfection, the antibody GMT was 197.8 U/mL (GSD 18.9), with an average time interval from the date of antibody determination to the onset of symptoms of 36.9 days (SD 41.1).

Of the 138 reports, 49 were unvaccinated, 14 were partially vaccinated, 70 were fully vaccinated, and 5 received 1 booster dose at the time of the report. The most common vaccine received was an inactivated vaccine (Sinovac) (in 43 cases or 31.2%, of which 40 completed the primary series while 3 received one heterologous booster vaccine dose). There were 49 cases (35.5%) who were unvaccinated, and 14 (10.2%) were partially vaccinated. Of the 5 participants who received a booster dose, 4 participants received mRNA Moderna booster and 1 participant received mRNA Pfizer booster. Only 6 cases adjudicated as unlikely reinfection (4.3%) had a history of exposure to a symptomatic close contact.

### 3.4 Association of SARS-CoV-2 antibody levels and development of reinfection

To determine the association of antibody titers on the development of probable reinfection, the hazards ratio was estimated using Cox proportional hazards model, with antibody titers taken as a continuous variable. The unadjusted hazard ratio (HR) was 0.994, 95% CI 0.992 to 0.996, p<0.001. Adjusting for age, sex, co-morbidities, use of immunosuppressants and vaccination status, the adjusted HR was similar at 0.994, 95% CI 0.992 to 0.996, p<0.001 (**Table 4**). In effect, for one unit increase in antibody titer, the risk of symptomatic reinfection decreased by 0.6%.For every 10 units increase in antibody titer, the risk of symptomatic reinfection decreased by 6%.

**Table 4:** Estimates of hazard ratio derived using the Cox proportional regression analysis.

Factor	Adjusted Hazards Ratio	95% Confidence Interval	P-value
Antibody, U/mL*	0.9939	0.9920 to 0.9958	<0.001
Vaccinated	1.2030	0.4574 to 3.1645	0.708
Sex	1.0321	0.6080 to 1.7520	0.907
Age, years	1.0118	0.9935 to 1.0305	0.209
Comorbidities, number			
1	1.8854	0.8180 to 4.3457	0.137
2 or more	0.9352	0.4981 to 1.7557	0.835

*Antibody level prior to reinfection for those with probable reinfection; antibody GMT for the 5 timepoints for those without probable reinfection. Unadjusted HR: 0.9939, 95% CI 0.9921 to 0.9959.

## 4 Discussion

### 4.1 Key findings

The total RBD-specific immunoglobulin levels in this study cohort increased over the one-year follow-up after natural SARS-CoV-2 infection. However, these results must be interpreted with caution since 288 study participants received varying types and doses of SARS-CoV-2 vaccine over the course of the study follow-up. With the high vaccination rate of the study cohort, an increase in the antibody levels is expected, as vaccination induces the production of anti-RBD binding and neutralizing antibodies (16).

We were able to observe the antibody levels of 11 study participants who remained unvaccinated for the entire 1-year follow-up period. In the subgroup of unvaccinated participants, the antibody titers also demonstrated an increase throughout the year. This result differs from the findings of other studies that reported a decline in IgG levels starting 6 months after natural SARS-CoV-2 infection (2, 5).

It is important to consider the epidemiologic context, particularly the timing of community surges of COVID-19 infection, in relation to the timing of the blood extractions. The Philippines experienced a surge of COVID-19 infection from the Delta variant from August to October 2021 and another surge from the Omicron variant from January to February 2022. The Delta variant surge coincided with the blood extraction for the third timepoint (Day 180) while the blood extraction for the fifth timepoint (Day 360) started during the peak of the Omicron variant surge. The timing of blood extractions in relation to the number of cases of COVID-19 in the Philippines is shown in **Figure 1**.

The 2.3-fold increase in antibody titers among the unvaccinated study participants at 6 months (day 180) compared to 3 months (day 90) may be explained by the surge of infections from the Delta variant in the community. These participants may have developed asymptomatic COVID-19 reinfection, which would cause an increase in the antibody titers. Studies also show that exposure to the SARS-CoV-2 virus may produce a mild increase in antibody titers, as observed among close contacts of COVID-19 patients who were not infected. However, this antibody response was observed to be more short-lived and declined more rapidly compared to those who developed the infection (17). A 4.9-fold increase in antibody titers was observed at 12 months (day 360) of follow-up compared to 9 months (day 270), which may be explained by the Omicron variant surge in the community. Similarly, asymptomatic reinfection or exposure to the SARS-CoV-2 virus may have caused the increase in the antibody titers of the unvaccinated study participants.

Population studies in other countries show different results. In China, the RBD-specific IgG, IgM and IgA antibodies were tested using an indirect electrochemiluminescence immunoassay kits (Kangrun Biotech Co., Ltd). There was a 2.87-fold decrease in RBD-IgG within 3 months (825 to 287 AU/mL), RBD-IgM decreased to negative levels within 3 months, and RBD-IgA became negative at 12 months,{2} In Lithuania, out of 38 study participants with quantitative SARS-CoV-2 S IgG levels measured using quantitative Enzyme Linked Immunosorbent Assay or ELISA (UAB Imunodiagnostika, Lithuania), 17 (44.7%) exhibited a decline in IgG levels from 6 months to 13 months, 14 (36.8%) had stable IgG levels, while 7 (18.5%) had increase in IgG levels (5). In Spain, a gradual decline was observed in S1 protein IgG antibodies detected through ELISA (Euroimmun AG, Lübeck, Germany) from 4 to 7 months (4).

The same laboratory test (Roche Elecsys^®^ Anti-SARS-CoV-2 S assay) was used in a seroprevalence study in South India. Authors reported an overall seroprevalence of 62.7% (95% CI 59.3 to 66.0), using 0.80 U/mL as the cut-off for a positive test. The case-to-undetected-infected ratio (CIR) was 1: 8.65 (95% CI 1:8.1 to 1:9.1) (18). Other studies that use the same laboratory test evaluated humoral responses to vaccination, not natural infection.

The results of this study also demonstrated that participants with more severe COVID-19 infection had significantly higher antibody titers compared to those with milder infection at day 21, consistent with the findings of other studies (2, 3, 5). The antibody titers persistently remained higher until day 180 among those with severe and critical infection, but the difference was no longer statistically significant.

The vaccination rate in this study cohort was high, with 90.5% participants who were fully vaccinated, and 69% receiving at least 1 booster dose. This rate was higher than the national rate of 77.8% for fully vaccinated individuals (as of June 2022) (19). Factors that may have led to a higher vaccine coverage in this cohort include better health seeking behavior as indicated by their willingness to participate in scientific research, and their residence in Metro Manila, which may lead to easier access to vaccine centers, Another possible factor is the frequent follow-up calls by the research team, where several participants would inquire about the safety and effectiveness of the vaccines. These calls provided good opportunities for the participant to express their concerns about the vaccines, and for the research team to clarify common misconceptions regarding vaccination.

All study participants demonstrated an increase in antibody titers regardless of initial disease severity classification and vaccination status. However, the participants who received SARS-CoV-2 vaccines had a greater rise in antibody levels compared to the unvaccinated group. This highlights the importance of vaccination even among previously infected individuals.

Studies that compared the antibody responses of vaccinated and naturally infected individuals report higher levels of anti-RBD or anti-S1 antibodies among those who received the vaccine (4, 20). These studies also reported a faster decline in antibodies among vaccinated individuals compared to those naturally infected, with one study reporting a decline in RBD antibodies within 6 months after vaccination, compared to 8 months for those naturally infected (20). In our study, we did not observe a decline in antibody levels for those unvaccinated and vaccinated individuals during the one-year follow-up.

In this study, there were 64 cases of probable reinfection. Due to the inaccessibility of genomic testing, reinfection could not be documented in this study. Instead, we estimated the prevalence of probable reinfection at 20.8%. Of the 64 participants with probable reinfection, only 1 was severe enough to necessitate hospitalization. This is consistent with the findings of other studies, which reported 90% lower odds of hospitalization or death for reinfections compared to primary infection. This is most likely due to the priming effect of the primary infection on the immune system, which enables a better immune response against the SARS-CoV-2 virus upon reinfection (21).

The 64 study participants with probable reinfection had an antibody GMT of 411.8 U/mL prior to the reinfection. Of the 64 study participants, 56 (87.5%) received the primary vaccine series and were considered fully vaccinated, while 5 of the 56 participants received a booster dose. Thus, these vaccinated participants could be classified as having breakthrough COVID-19 infection as well. Reinfection occurred at 143 days on average (range 13 to 236 days) after completing the primary vaccine series. Among those who remained unvaccinated, reinfection occurred on average 198 days after the initial infection or the positive RT-PCR test result for asymptomatic patients.

In this study, increased antibody levels were found to be significantly associated with reduced symptomatic reinfection rate (p<0.001). This is consistent with other studies that reported on the correlation of high levels of anti-RBD IgG with a reduced risk of symptomatic infection (22). Although there is no well-defined cut-off for the antibody level that confers protection against COVID-19 infection, one study involving fully vaccinated participants reported 80% vaccine efficacy against symptomatic infection with the alpha variant of SARS-CoV-2 with anti-RBD immunoglobulin levels of 506 binding antibody unit (BAU)/mL (95% CI 135 to beyond data range) (17). These findings highlight the importance of vaccination, and the potential of properly timed booster doses to enhance the protective immune response.

It can also be observed in this study that the study participant with severe reinfection had lower antibody titers compared to those with mild or moderate reinfection. This finding is consistent with the results of a cohort study in South Korea wherein antibody levels were found to predict the clinical course of patients with delta and omicron variant COVID-19 infection. Those with increased antibody levels had decreased occurrence of fever, hypoxia, CRP elevation, and lymphopenia (23).

### 4.2 Limitations

The limitations of this study include the focus of the study on determining antibody titers and its correlation with protection against future infection. Recent studies have highlighted the major role of T cells in developing immunity against SARS-CoV-2. T cells have been observed to last for at least 6 months after natural infection. T cells were also observed to increase upon exposure to low-dose SARS-CoV-2 virus, leading to the hypothesis that memory T cells may provide protection against severe reinfection (24).

Another limitation is that the study involved the measurement of binding antibodies (anti-RBD antibodies) and not neutralizing antibodies. Neutralizing antibodies play a critical role in protecting against SARS-CoV-2 by clearing the virus. Neutralizing antibodies interfere with the binding of the virus to its receptor, block the uptake of virus into host cells, and prevent the uncoating of viral genomes (25). Levels of neutralizing antibodies are highly predictive of immune protection from SARS-CoV-2 infection (26). Measurement of neutralizing antibodies against SARS-CoV-2 utilizing the plaque reduction neutralization test (PRNT) was originally planned since this is the reference standard. The test, which measures the level of neutralizing antibodies, is tedious and takes 4 to 5 days to complete. The procedure typically requires the use of live virus, using a specialized set-up in a biosafety level 3 (BSL3) laboratory (13, 27). At the time of study implementation, there was no certified BSL3 laboratories in the country. However, several studies report that neutralizing and anti-RBD IgG antibody levels are strongly correlated, and that anti-RBD IgG antibody levels can be used for the accurate assessment of immunity following SARS-CoV-2 infection or vaccination (4, 28, 29).

It is also important to note that amino acid substitutions in the RBD of the different COVID-19 variants may affect the binding of antibodies (30). Furthermore, only 1 immunoassay was used in this study. Correlation of antibody titer results with other immunoassays could not be done.

In this study, the laboratory test used had a lower limit of detection of 0.40 U/mL and an upper limit of detection of 250 U/mL. As per manufacturer recommendations, 10-fold dilution was performed to increase the upper limit of detection to 2,500 U/mL. However, several results still exceeded 2,500 U/mL. Further 100-fold and 1,000-fold dilutions were done to increase the upper limit of detection to 250,000 U/mL. The accuracy of the test may have diminished at these higher range of values.

Another limitation in this study is the variation in interval between determination of antibody GMT levels and the development of reinfection. Determination of antibody GMTs was performed at fixed time points based on the time of initial diagnosis of COVID-19 regardless of the time of diagnosis of reinfection or the time of vaccination.

Furthermore, due to limitations in the study funding, testing *via* RT-PCR or antigen test was encouraged but not provided for free for the study participants. Some study participants who developed symptoms consistent with a COVID-19 reinfection refused to undergo testing. We identified 101 reports of possible reinfection in the study cohort that could be true reinfections; however, the lack of supportive tests preclude definite classification. The study was also unable to detect cases of asymptomatic reinfection. Thus, the number of cases of reinfection reported in this study may be underestimated.

### 4.3 Contribution to knowledge and future research implications

This study observed an increase in antibody levels over the study period among both unvaccinated and vaccinated patients previously infected with COVID-19 infection residing in Metro Manila, Philippines. These data contribute to knowledge on the long-term humoral immune response to COVID-19, which is affected by severity of initial disease, vaccination, COVID-19 reinfection, and exposure to new variants during the Delta and Omicron surges. It also demonstrates that higher antibody levels are associated with a lower risk of symptomatic reinfection. Future research that monitor levels of neutralizing antibodies against specific COVID-19 variants, as well as research on the levels of neutralizing antibody among patients who develop definite asymptomatic and symptomatic re-infection may be done.

## 5 Conclusion

This cohort study demonstrated an increase in antibody levels against SARS-CoV-2 over one year among those who had COVID-19 infection. Several factors could have led to the steady increase in antibody levels, including COVID-19 vaccination, COVID-19 reinfection, and exposure to new variants during the Delta and Omicron surges. Participants with more severe COVID-19 infection had significantly higher antibody levels compared to those with milder infection at day 21. There were 64 cases of probable reinfection identified in the study cohort, of which 56 (87.5%) were fully vaccinated. Higher antibody levels were associated with a lower risk of symptomatic reinfection. Information on the timing, magnitude, and durability of humoral immunity among Filipinos is essential to guide the deployment of vaccine stocks, and can help guide strategies for returning to the workplace and relaxing social distancing measures.

## Data availability statement

The raw data supporting the conclusions of this article will be made available by the authors, without undue reservation.

## Ethics statement

The studies involving human participants were reviewed and approved by University of the Philippines Manila Research Ethics Board. The patients/participants provided their written informed consent to participate in this study.

## Author contributions

All authors contributed to conception and design of the study. MD, CT-L organized the database. CT-L, CC performed the statistical analysis. CT-L wrote the first draft of the manuscript. All authors contributed to manuscript revision, read, and approved the submitted version.
